# Spices and Herbs Increase Vegetable Palatability Among Military Service Members

**DOI:** 10.1093/milmed/usae367

**Published:** 2024-07-30

**Authors:** Christopher R D’Adamo, Melissa R Troncoso, Gabriela Piedrahita, Joshua Messing, Jonathan M Scott

**Affiliations:** Department of Family and Community Medicine, University of Maryland School of Medicine, Baltimore, MD 21201, USA; Nova Institute for Health, Baltimore, MD 21231, USA; Navy Medicine Readiness and Training Command, Naval Medical Center Portsmouth, Portsmouth, VA 23708, USA; Nova Institute for Health, Baltimore, MD 21231, USA; Department of Family and Community Medicine, University of Maryland School of Medicine, Baltimore, MD 21201, USA; Consortium for Health and Military Performance, Department of Military and Emergency Medicine, F. Edward Hebert School of Medicine, Uniformed Services University of the Health Sciences, Bethesda, MD 20814, USA

## Abstract

**Introduction:**

Unhealthy eating behaviors are adversely impacting the health and performance of the U.S. armed forces. Vegetable intake, in particular, has been shown to be far below recommended levels in active duty military populations. Previous research in other populations has shown that the addition of spices and herbs can help overcome numerous barriers to vegetable intake. The goal of this study was to determine modifiable barriers to vegetable intake among a sample of active duty military service members at Naval Support Activity Bethesda and evaluate whether the addition of spices and herbs can help surmount these barriers.

**Materials and Methods:**

Monadic sensory testing was conducted that compared typical preparation (butter and salt) of 4 vegetables (broccoli, carrots, cauliflower, and kale) vs. otherwise identical preparation with the addition of spices and herbs. The Menu Item Survey, a 9-point hedonic scale utilized throughout the U.S. Military for recipe development, was the primary outcome of the vegetable sensory testing. Questionnaires were administered to assess barriers to military dining facility vegetable intake. Unpaired *t*-tests were utilized to compare Menu Item Survey ratings of typical vegetables vs. vegetables with spices and herbs. Descriptive statistics were computed to summarize the results of the barriers questionnaires, and qualitative analysis of open-ended questions was conducted to identify perceived areas of improved vegetable intake.

**Results:**

A diverse sample of 70 active duty service members participated in the vegetable sensory testing and provided outcome data. The most common barriers to military dining facility vegetable intake were appearance (42.9%), preparation style (41.3%), and taste (39.7%). Sensory testing revealed that vegetables with spices and herbs were preferred over typical preparations in overall appeal, flavor, and aroma (*P* <.03).

**Discussion:**

The addition of spices and herbs appears to help overcome key sensory-related barriers to military dining facility vegetable intake. Future comparison of vegetable intake with and without spices and herbs when included in a full meal in a military dining setting is warranted in order to better evaluate the effectiveness in increasing vegetable intake under typical dining conditions.

## INTRODUCTION

Unhealthy eating behaviors among the U.S. armed forces is an area of growing concern. Military service members have consistently reported consuming diets that are widely discordant with the recommendations of the USDA Dietary Guidelines for Americans (DGA).^1–4^ Vegetable intake, in particular, has been revealed as lacking, with just 12.9% of active duty military meeting the vegetable intake recommendations of the DGA in recent analysis. Among other diet-related health concerns, the 2018 Health-Related Behaviors Survey of the U.S. DoD revealed a prevalence of obesity of 15.1% among active duty military personnel.^5^ The prevalence of obesity varies widely among the branches of the armed forces, ranging from 8.3% among the Marines to over 22% among the Navy.^6,7^ Furthermore, these data revealed that only 33.3% of active duty service members were at a normal weight. In addition to the high prevalence of obesity and overweight, poor diet quality has been shown to adversely affect the psychological resilience^8^ and physical performance^9,10^ of military personnel. Collectively, these issues have contributed to the well-documented decreases in the readiness and ability to meet the intense demands of military life.

The well-established physical and psychological effects of poor diet quality among military service members have led to increasing study into the root causes of unhealthy eating behaviors among the 1.3 million active duty service members of the U.S. armed forces.^11^ The food environment on and surrounding many military bases appears to be a major contributor to poor diet quality in this demographic.^12^ A study among participants in the U.S. Army Office of the Surgeon General’s Worksite Health Promotion Program revealed that soldiers perceived the military campus-style food environment as inhibitory to healthy decision-making and an impediment to healthy eating.^13^ Service members described the proximity and density of fast-food restaurants and relative lack of access to and higher cost of healthy foods on the base as barriers to consuming vegetables and other healthy foods. Recent investigation into factors that influence eating behaviors among sailors across multiple U.S. Navy bases also concluded that the military food environment contributed to unhealthy eating behaviors.^14^ In addition, the pressure to eat on the go because of mission-first culture encouraged sailors to eat fewer nutritious foods. Numerous factors supporting healthy eating behaviors among service members were also identified in this study, which included access to healthy, convenient, and low-cost foods on the base.

In response to these and other findings, broad efforts have recently been made to improve the eating behaviors of military personnel. These efforts have included direct intervention in the food served at military dining facilities. The Special Operations Forces Human Performance Program military dining facility nutrition intervention focused primarily on food as a means of performance optimization and included menu modifications to increase daily offerings of nutrient-dense, performance-oriented foods.^15^ The intervention resulted in higher Healthy Eating Index scores and dining facility satisfaction compared with the control condition (60.1 vs. 49.0, respectively, *P* = .005). Vegetable intake was also improved, with the most notable improvement found in the intake of red and orange vegetables. Similarly, a menu modification intervention at the U.S. Army dining facilities revealed improvements in both diet quality and satisfaction with the flavor of the food.^16^ These data suggest that improved access to healthier, more flavorful foods may improve the eating behaviors of military service members.

Although not yet studied in a military population, the potent sensory-engaging properties of spices and herbs appear to make them a promising intervention for overcoming barriers to healthy eating and improving diet quality in this population. Previous work has shown that spices and herbs can be effective vehicles for improving eating behaviors among populations with low diet quality, including racially and geographically diverse samples of high school students in urban environments,^17–19^ rural environments,^20^ and university populations.^21–23^ These findings in younger populations offer promise as more than half of active duty enlisted military service members are between 18 and 25 years of age.^24^

Although spices and herbs have demonstrated potential in overcoming barriers to vegetable consumption in younger populations, there have been no studies conducted to date evaluating whether they are effective in a military dining setting. There were 2 related primary goals of this study. The first goal was to identify potentially modifiable barriers to consuming vegetables among military service members specifically while living and/or working on a large military base. The second goal was to utilize monadic sensory testing to evaluate whether the addition of spices and herbs to vegetables served to military service members is preferable to typical vegetable offerings that were otherwise identical (e.g., same amounts of fat, sugar, and salt) without spices and herbs. The research team hypothesized that there would be numerous modifiable barriers to vegetable intake on the military base and that sensory testing would reveal that most vegetables with spices and herbs would be deemed preferable to the typical vegetable preparations offered on the base.

## METHODS

### Study Design

A multimethod outcomes evaluation consisting of quantitative and qualitative questionnaire data and sensory testing of vegetables with and without spices and herbs was conducted among a sample of military service members on a large military base in the United States. The study received expedited approval by the Institutional Review Boards of the Uniformed Services University (approval no: 948,662) and the University of Maryland School of Medicine (approval no.: HP-00095407). Informed consent was not required by the Institutional Review Boards that approved the study because of the minimal risk of the intervention and the de-identified nature of all data that were collected. The study was registered on ClinicalTrials.gov (NCT05499858).

### Study Setting

The study was conducted at Naval Support Activity Bethesda (NSAB), a military base located in Bethesda, Maryland, that includes Walter Reed National Military Medical Center, the Uniformed Services University (USU), and the Warrior Transition Brigade and supports more than 40 tenant commands. There are a variety of food establishments on the base, including a convenience store, several fast-food restaurants, Warrior Café—(military dining facility), and the USU cafeteria and snack shop. The sensory testing and data collection were performed at a United Service Organization located between 2 barracks, Warrior Transition Unit and the USU cafeteria.

### Study Sample

Study volunteers were required to be at least 18 years of age, in the military with a rank of E1-E4 or O1-O3, and able to read and write in English. Study participants were recruited throughout NSAB via word of mouth, printed flyers, and communication with key leaders and other stakeholders.

### Intervention

The research team worked with a variety of key stakeholders throughout the NSAB community to identify ways to best assess potential barriers to vegetable intake in this military population. The stakeholders with whom we collaborated included military service members, staff dietitians, the health promotion specialist on the base, barracks managers, military culinary specialist, unit leaders, morale welfare and recreations/single sailor program leaders, base senior enlisted leaders, and the base commander. Information obtained in the stakeholder engagement process informed logistics of the intervention implementation (location, timing, etc.), participant recruiting strategy, specific vegetables to be studied, and the vegetable recipes currently served in military dining facilities to which we would be comparing the new vegetable recipes with the addition of spices and herbs.

In order to isolate the inference of the addition of spices and herbs to vegetables in the sensory testing process, the vegetable recipes with spices and herbs were prepared in the same manner and contained the same ingredients as the typical vegetable recipes with the exception of the added spices and herbs. The vegetables analyzed in the sensory testing process were broccoli, carrots, cauliflower, and kale. The typical preparation of these vegetable recipes included added salt and compound butter. In addition to the same amounts of salt and compound butter, Supplementary Table S1 lists the spices and herbs that were used for each of the vegetables under study. In brief, the flavor profiles of the vegetables with spices and herbs were savory broccoli (most abundant herbs and spices—garlic powder, onion powder, black pepper, and mustard seed), curry cauliflower (most abundant herbs and spices—garlic powder, onion powder, turmeric, and coriander), sweet spice carrots (most abundant herbs and spices—vanilla and cinnamon), and smoky kale (most abundant herbs and spices—garlic powder, onion powder, paprika, and oregano). With an eye toward potential reproducibility, the recipes included only widely available and relatively affordable spices and herbs that could potentially be applied in other military dining settings if so desired. All vegetables that were compared in the sensory testing were initially steamed, as stakeholder engagement revealed that this was the most common method of preparation in most military dining facilities. The steamed vegetables were then frozen along with a frozen compound butter containing butter and salt (typical recipes) or butter and salt with spices (spiced recipes) that was placed on top of each vegetable. The frozen vegetables were microwaved, which melted the compound butter into the vegetables. The vegetables were also briefly stirred by a research associate before serving, to help further ensure even distribution of the compound butter across the vegetable being served. All participants underwent the crossover with every vegetable. Although blinding was not possible because of the differences in both appearance and aroma between the vegetables with and without spices and herbs, the order in which the vegetables were served to all participants was randomized to minimize expectation bias (e.g., spiced always served first or typical always served first). A washout period of several minutes in between vegetable servings was implemented. The washout consisted of providing table crackers and water, which were consumed by participants in between each vegetable testing to clear their palate before the next vegetable testing. The vegetable testing was conducted at both lunch (approximately 11:00 am–1:00 pm) and dinner (approximately 4:00–6:00 pm) to provide flexibility and to accommodate the busy schedules of the participating service members.

### Outcomes

The participants completed questionnaires assessing a variety of modifiable barriers to consuming military dining facility vegetables and the importance of a variety of characteristics of the vegetable preparations. The participants also completed a questionnaire used in previously published research^19^ describing their familiarity, liking, and current usage of a wide variety of spices and herbs. Frequencies were reported for the barriers to military dining facility vegetable intake. Ratings on various characteristics of the vegetables currently served in military dining facilities were assessed with a 5-point Likert scale (higher scores indicating more favorable ratings), and the importance of the characteristics of the vegetables served in this setting was assessed with a 3-point Likert scale (higher scores indicating greater importance). Qualitative data were collected regarding general impressions on military dining vegetables and ways in which participants expressed that they could be improved.

Outcomes of the sensory testing process of the vegetables with and without spices and herbs were assessed using the U.S. Military—Menu Item Survey. This survey, provided in the Supplementary Material, is used by the Combat Feeding Division, Armed Forces Recipe Services when developing and evaluating new recipes to be used in military dining facilities around the world. The survey is used to evaluate the sensory properties of menu items. In brief, the survey contains a traditional 9-item hedonic scale rating of liking, specific ratings of the appearance, aroma, flavor, texture, and overall appeal of the menu item. The survey is scored from 1 (dislike extremely) to 9 (like extremely). Qualitative feedback was also collected assessing general impressions of the menu item. Each study participant provided ratings of the overall liking, sensory characteristics, and qualitative feedback for each vegetable with and without spices and herbs. This design was utilized to minimizing the potential for confounding by participant characteristics (e.g., taste preferences and previous exposures) that could have occurred with a parallel-arm design.

### Statistical Analysis

Descriptive statistics (means, frequencies, etc.) were computed to characterize the demographics of the study sample, the ratings of the characteristics of the vegetables currently being served military dining facilities, the importance of those characteristics, and spices and herbs. Paired *t*-tests were utilized to compare overall liking and specific sensory characteristics between the vegetables with and without spices and herbs. Qualitative data were reviewed, categorized, and ranked according to the frequency of responses. Statistical significance was defined as *P* <.05. All statistical analyses were conducted in SAS Version 9.4.1 (Cary, NC).

### Copyright Statement

This work was prepared by a military or civilian employee of the U.S. Government as part of the individual’s official duties and therefore is in the public domain and does not possess copyright protection. Title 17 U.S.C. 105 provides that “copyright protection under this title is not available for any work of the United States Government.” Title 17 U.S.C. 101 defines a U.S. Government work as work prepared by a military service member or employee of the U.S. Government as part of that person’s official duties. (Public domain information may be freely distributed and copied; however, as a courtesy, it is requested that the Uniformed Services University and the authors be given an appropriate acknowledgment).

## RESULTS

Seventy participants participated in the vegetable sensory testing and provided outcomes data. The study sample was diverse with respect to ethnicity (37.1% Caucasian, 18.6% Hispanic, 17.1% African American, 14.3% Asian American, and 12.3% mixed/other), gender (54.3% male and 45.7% female), and rank (62.3% officer and 37.7% enlisted) and had a mean [SD] age of 27.4 [5.5] years.

The most commonly reported barriers to vegetable intake in military dining facilities were as follows: appearance (42.9%), preparation style (41.3%), taste (39.7%), texture (31.8%), disliking the types of vegetables served (23.8%), and aroma (18.8%). Social factors did not appear to be meaningful barriers to military dining facility vegetable intake, with no previous exposure to the types of vegetables served (7.9%), insufficient time (3.2%), and friends not eating the vegetables (1.7%) being reported infrequently. Qualitative analysis revealed that the most commonly reported suggestions to increase the intake of vegetables in the military dining facilities were to improve the preparation style (*n* = 17), flavor (*n* = 12), and variety (*n* = 11) of the vegetables.

The mean [SD] ratings of the characteristics of the vegetables currently being served in the military dining facilities vegetable characteristics on a 5-point Likert scale are as follows: taste = 3.36 [0.71], appearance = 3.33 [0.74], variety = 3.45 [0.73], and how healthy they are = 3.72 [1.0].

The mean [SD] importance of the vegetable characteristics on a 3-point Likert scale are as follows: taste = 2.73 [0.45], appearance = 2.32 [0.67], variety = 2.57 [0.55], and how healthy they are = 2.62 [0.58].

The 3 most familiar spices and herbs were garlic powder (100%), black pepper (97.1%), and oregano (97.1%). The 3 least familiar spices and herbs were celery seed powder (43.2%), cloves (65.7%), and allspice (66.2%). The 3 most liked spices and herbs were garlic powder (100%), onion powder (100%), and black pepper (100%). The 3 least liked spices and herbs were celery seed (83.1%), sage (88.1%), and nutmeg (89.2%). The 3 most commonly reported spices and herbs as favorites were garlic powder (27.9%), onion powder (22.1%), and black pepper (14.9%). The most commonly reported foods with which spices and herbs were consumed were chicken (*n* = 105), pasta (*n* = 105), and pizza (*n* = 49). The number of commonly reported foods with which spices and herbs were consumed exceeds the number of study participants because multiple preparations were permitted in these open-ended assessments.

**Tables 1** **–4** provide the sensory testing ratings comparing each vegetable (broccoli, carrots, cauliflower, and kale) with and without spices and herbs.

**Table 1.** Menu Item Survey sensory testing scores for typical broccoli vs. broccoli with spices and herbs (*n* = 70)^a^.

	Typical	Spices and Herbs	*P*-value^b^
Overall appeal	6.75 ± 1.51	7.42 ± 1.15	<.0001
Appearance	6.86 ± 1.55	6.83 ± 1.47	.8
Aroma	6.36 ± 1.64	7.21 ± 1.37	<.0001
Flavor	6.37 ± 1.67	7.47 ± 1.28	<.0001
Texture	6.61 ± 1.82	7.0 ± 1.35	.03

a Mean 9-point Likert scale ratings with ± indicating SD.

b *P*-values determined through comparison of means with paired *t*-tests.

**Table 2.** Menu Item Survey sensory testing scores for typical carrots vs. carrots with spices and herbs (*n* = 70)^a^.

	Typical	Spices and Herbs	*P*-value^b^
Overall appeal	6.49 ± 1.64	7.28 ± 1.42	.0002
Appearance	6.76 ± 1.55	7.2 ± 1.36	.01
Aroma	5.94 ± 1.60	7.46 ± 1.52	<.0001
Flavor	6.57 ± 1.64	7.26 ± 1.58	.002
Texture	6.28 ± 2.06	6.59 ± 2.07	.1

a Mean 9-point Likert scale ratings with ± indicating SD.

b *P*-values determined through comparison of means with paired *t*-tests.

**Table 3.** Menu Item Survey sensory testing scores for typical cauliflower vs. cauliflower with spices and herbs (*n* = 70)^a^.

	Typical	Spices and Herbs	*P*-value^b^
Overall appeal	5.69 ± 1.76	6.78 ± 1.20	<.0001
Appearance	5.60 ± 1.56	6.40 ± 1.50	.002
Aroma	5.17 ± 1.74	6.94 ± 1.41	<.0001
Flavor	5.58 ± 1.87	6.19 ± 1.49	.03
Texture	6.37 ± 1.81	6.7 ± 1.4	.046

a Mean 9-point Likert scale ratings with ± indicating SD.

b *P*-values determined through comparison of means with paired *t*-tests.

**Table 4.** Menu Item Survey sensory testing scores for typical kale vs. kale with spices and herbs (*n* = 70)^a^.

	Typical	Spices and Herbs	*P*-value^b^
Overall appeal	5.59 ± 1.93	5.82 ± 1.88	.3
Appearance	6.3 ± 1.76	6.16 ± 1.75	.5
Aroma	5.53 ± 1.92	6.1 ± 1.83	.02
Flavor	5.15 ± 2.08	5.64 ± 2.24	.07
Texture	5.55 ± 2.07	5.59 ± 2.10	.8

a Mean 9-point Likert scale ratings with ± indicating SD.

b *P*-values determined through comparison of means with paired *t*-tests.

The overall appeal reported for the broccoli, carrots, and cauliflower with spices and herbs was greater than for the typical (butter and salt) vegetables (*P* <.0002). The percentage difference in overall appeal of these vegetables with spices and herbs ranged from 9.92% higher (broccoli) to 19.2% (cauliflower) higher than the typical vegetables. There was no difference in overall appeal between the typical kale and the kale with spices and herbs (*P* = .3).

Aroma was the sensory dimension that was most impacted by the addition of spices and herbs. The aroma of the vegetables with spices and herbs was preferable to the aroma of the typical preparations for all vegetables under study (*P* <.03). The percentage difference in aroma of the vegetables with spices and herbs ranged from 10.3% higher (kale) to 34.2% higher (cauliflower) than the typical vegetables.

The flavor reported for the broccoli, carrots, and cauliflower with spices and herbs was greater than for the typical vegetables (*P* <.03). The percentage difference in flavor of these vegetables with spices and herbs ranged from 10.5% higher (carrots) to 17.3% higher (broccoli) than the typical vegetables. There was no statistically significant difference in flavor reported between the typical kale and the kale with spices and herbs (*P* = .07).

The appearance of the carrots (6.5%, *P* = .01) and cauliflower (14.3%, *P* = .002) with spices and herbs was preferable to the typical preparations. There was no difference in appearance reported for broccoli (*P* = .8) and kale (*P* = .5).

Texture was the sensory domain least impacted by the addition of spices and herbs. The percentage difference reported in the texture of the cauliflower (5.2%, *P* = .046) and broccoli (5.9%, *P* = .03) was higher for the vegetables with spices and herbs than for the typical vegetables. There was no difference in texture reported between the carrots (*P* = .1) and kale (*P* = .8) with spices and herbs and the typical preparations.

## DISCUSSION

This was the first study to the authors’ knowledge aimed at determining whether spices and herbs might be able to surmount modifiable barriers to vegetable intake within the dining facilities of a large U.S. Military base. There are over 4,800 U.S. Military sites in over 160 countries worldwide serving tens of thousands of meals per day to the military service members, whose intake of vegetables has been consistently shown to be below recommended levels. Although the food and social environments in many military bases pose challenges to healthy eating, the specific barriers to vegetable intake in this setting uncovered in this study offer some encouragement. Social and environmental factors, such as limited time because of the demands of military life and lack of previous exposure to vegetables, were not commonly reported barriers to vegetable intake on the military base. Some of the most commonly reported barriers of vegetable appearance, flavor, and aroma are modifiable with the addition of widely available and inexpensive spices and herbs.

The results of the sensory testing revealed that vegetables with spices and herbs had greater overall appeal, aroma, and flavor than the typical preparations without spices and herbs for most of the vegetables under study. Aroma was the domain most consistently associated with higher ratings with the addition of spices and herbs. Not surprisingly, texture was the domain with the lowest difference in ratings with the addition of spices and herbs, as the majority of the spices and herbs added to the vegetables in this study were powders with minimal impact on texture. The addition of spices and herbs had a greater impact on the appearance of lighter colored vegetables (cauliflower and carrots) than the green vegetables (kale and broccoli) under study. This was also not surprising, because of less visual contrast in appearance with darker colored vegetables. Neither the overall appeal nor any specific domain of the sensory testing revealed a preference for typical preparations of any of the vegetables under study, suggesting consistent preference for the vegetables with spices and herbs.

There were several key strengths of this study. First, the vegetable preparations with and without spices and herbs that were compared in the sensory testing were identical other than the spices and herbs content. Consistent amounts of salt, fat, and sugar in the vegetable preparations under comparison minimized the potential for confounding by dietary components other than spices and herbs. Another strength was the extensive military stakeholder engagement process implemented in this study, which enabled the successful recruitment of a diverse sample of military personnel spanning ethnicity, gender, and rank. Informed by the stakeholder engagement process, the vegetable intake barrier questionnaires and sensory testing were conducted in locations on base where the military service members typically congregate and commonly consume their meals, which helped facilitate recruitment of a diverse sample. The diversity of the sample enhances the generalizability of the encouraging findings in this study. Finally, the outcome measure for the vegetable sensory testing was the Menu Item Survey, which is used widely across the U.S. Military to evaluate recipes served in military dining facilities. This survey was chosen to evaluate the overall appeal and specific sensory domains of the vegetable preparations under study as it enables a common frame of comparison for current military vegetable recipes and fosters reproducibility in other military settings.

There were also a number of notable limitations to this study. Although the order of vegetables served in the sensory testing was randomized to reduce expectation bias, blinding was not possible because of the discernible differences in aroma and appearance of most of the vegetables that were compared. Although the order of vegetables served was randomized, the study participants were not enrolled from a random sample. This introduced the potential for selection bias and limits extrapolation. While the preference for the spiced recipes vs. typical recipes was consistent across the vegetables under study, the absolute difference in preferences was generally relatively modest. All vegetables under study were steamed before reheating in order to align with the simplest, most common preparation method and support the potential for reproducibility in other settings. Additional studies would be required to determine if there was a similar preference for spices under other vegetable preparation methods (e.g., sauteing and roasting). A final limitation is that the vegetables were served alone. This was deemed preferable to isolate sensory properties of the vegetables under comparison and was the most feasible option given the study setting, but the real world effects comparing vegetable preferences with and without spices and herbs when offered as part of a complete meal also warrant future study.

## CONCLUSIONS

Spices and herbs appear to be promising tools to help overcome several common barriers to vegetable intake in military dining facilities. Although vegetables with added spices and herbs were consistently preferred to the otherwise identical typical vegetable preparations in this study, the addition of spices and herbs appears to be a promising component of a more comprehensive approach to healthier eating, including enhanced access, education, and environmental modification. In order to better evaluate the effectiveness under typical dining conditions, future directions of this research include comparison of vegetable intake with and without spices and herbs when included in a full meal in a military dining setting.

## Supplementary Material

usae367_Supp

## Data Availability

The raw data from this study are publicly available at Figshare (10.6084/m9.figshare.25557078).
