# Supplementary material for: Spices and Herbs Increase Vegetable Palatability Among Military Service Members
Source: Mil Med. 2024 Jul 30;190(1-2):e266–72. doi: 10.1093/milmed/usae367 (PMC11737314; doi:10.1093/milmed/usae367)
Supplement: usae367_Supp [file usae367_supp.zip › Supplemental Table 1 - Military Vegetables Spices and Herbs.docx]

**Supplemental Table 1:**

**Spices and Herbs Contained in Vegetable Recipes Evaluated in Sensory Testing**

| **Vegetable** | **Spices and Herbs*** |
| --- | --- |
| Broccoli | Garlic powder, onion powder, black pepper, ground mustard seed, ground cumin, parsley flakes, dill weed, cayenne |
| Carrots | Vanilla extract, ground cinnamon |
| Cauliflower | Garlic powder, onion powder, ground coriander, ginger  turmeric, ground cumin, black pepper, ground mustard seed, chili powder, ground cinnamon, cayenne |
| Kale | Garlic powder, onion powder, smoked paprika,  oregano, black pepper, ground cumin, crushed red pepper |

* Spices and herbs listed in order from highest to lowest amounts contained
